# Post-translational Modification Control of HBV Biological Processes

**DOI:** 10.3389/fmicb.2018.02661

**Published:** 2018-11-01

**Authors:** Fan Yang

**Affiliations:** State Key Laboratory for Diagnosis and Treatment of Infectious Diseases, The First Affiliated Hospital, School of Medicine, Zhejiang University, Hangzhou, China

**Keywords:** HBV, post-translational modifications, cccDNA, replication, transcription, hepatocarcinogenesis

## Abstract

Hepatitis B virus infection remains a global healthy issue that needs to be urgently solved. Novel strategies for anti-viral therapy are based on exploring the effective diagnostic markers and therapeutic targets of diseases caused by hepatitis B virus (HBV) infection. It is well-established that not only viral proteins themselves but also key factors from the host control the biological processes associated with HBV, including replication, transcription, packaging, and secretion. Protein post-translational modifications (PTMs), such as phosphorylation, acetylation, methylation, and ubiquitination, have been shown to control protein activity, regulate protein stability, promote protein interactions and alter protein subcellular localization, leading to the modulation of crucial signaling pathways and affected cellular processes. This review focuses on the functions and effects of diverse PTMs in regulating important processes in the HBV life cycle. The potential roles of PTMs in the pathogenesis of HBV-associated liver diseases are also discussed.

## Introduction

Despite the available vaccine against hepatitis B virus (HBV) developed in 1981, there are still 364 million people worldwide chronically infected with this hepatotropic virus (Polaris Observatory Collaborators, 2018). The pathogenesis of various severe liver diseases, such as hepatocellular carcinoma, fibrosis, cirrhosis, and acute liver failure, are associated with persistent HBV infection (Lavanchy, 2004; Torres and Davila, 2012; Wu et al., 2018). High morbidity and mortality are attributed to chronic HBV, although the numbers have been decreasing over the past years (Global Global Burden of Disease Study 2013 Collaborators, 2015). Interferons (IFN) and nucleos(t)ide analogs (NAs) are currently the main therapeutic regimens, and the inability to eliminate the virus is attributed not only to drug resistance and side effects but also to the difficulty in eliminating covalently closed circular DNA (cccDNA) and the viral genome integrated in chromosomes (Torres and Davila, 2012). Thus, HBV infection remains a critical global healthy problem that requires the development of novel strategies for anti-HBV therapy.

Hepatitis B virus is a member of the Hepadnaviridae family and mainly consists of its viral envelope and an icosahedral nucleocapsid core with a partially double strand relaxed circular DNA (rcDNA) (Seeger and Mason, 2000). The rcDNA bound with the viral polymerase is released from the capsid, enters into the nucleoplasm and then converts into a cccDNA during infection (Nassal, 2015). The cccDNA, which is formed by a nucleosome-bound minichromosome, is the template for viral messenger RNA (mRNA) transcript synthesis (Bock et al., 2001). The stable existence of cccDNA in infected hepatocytes is one of the key factors for HBV persistent infection.The template for HBV genome replication is pre-genomic RNA (pgRNA), which is one of the transcripts synthesized from the cccDNA (Beck and Nassal, 2007). The viral minus DNA strand is then synthesized from a pgRNA template and serves as the template for the plus DNA strand in the core particle (Seeger and Mason, 2000). After enveloping, some mature core particles are released and others are cycled back to the nucleus (Levrero et al., 2009; Koumbi and Karayiannis, 2015). Four overlapping open reading frames (ORFs) that are partially encoded by the 3.2-kb HBV genome are ultimately translated into necessary proteins, including the HBV core antigen (HBcAg), HBV surface proteins (HBs), HBV X proteins (HBx) and other polymerases, for virus survival (Seeger and Mason, 2000). The regulation of viral replication, transcription and translation not only relies on various viral elements but also depends on many host factors (Moolla et al., 2002).

Post-translational modifications (PTMs) are critical for regulating signal molecule activity in different pathways, controlling protein stability and promoting the interaction between ligands and receptors in various cellular processes (Deribe et al., 2010). Diverse PTMs are known to play a vital role in regulating viral biological processes according to their functions in viral survival and proliferation (Zhang et al., 2013; Chang et al., 2016). Classical PTMs (such as phosphorylation and glycosylation) and increasingly regarded PTMs (such as acetylation, methylation, ubiquitination, SUMOylation, and NEDDylation) have been proven to influence HBV replication, transcription, and immune evasion via their modified targets including viral essential proteins and host-related components, such as transcription factors (Pollicino et al., 2006; Bai et al., 2015; Selzer et al., 2015; Liu et al., 2017). PTMs also control processes in which HBV is deemed to be the major cause of pathogenesis, such as several liver diseases, including hepatocarcinogenesis (Zhang et al., 2016; Gao et al., 2017). In this review, we summarize the positive and negative regulation effects of PTMs in viral biological processes and their roles in the pathogenesis of HBV-associated liver diseases.

## The Role of Phosphorylation in the Regulation of HBV Life Cycle

Phosphorylation is one of the most common PTM types in the viral life cycle, and it is regulated by various kinases and phosphatases. Viral protein phosphorylation and dephosphorylation plays a key role in regulating viral activity. The HBV core protein, which is composed of 183 to 185 amino acid residues, can be divided into an N-terminal domain (NTD) for capsid shell formation, a C-terminal domain (CTD) that is essential for HBV replication, and a linker between the NTD and CTD that is crucial for pgRNA packaging, viral DNA synthesis and virion secretion (Yu and Summers, 1991; Nassal, 1992; Liu et al., 2018). The HBc CTD is highlighted for containing several important serine and threonine phosphorylation sites in viral replication (Melegari et al., 2005; Lewellyn and Loeb, 2011; Jung et al., 2014). Three major serine phosphorylation sites (S155, S162, and S170) have been reported for their roles in producing HBV DNA replicative intermediates and rcDNA intermediates in an HBx-dependent manner by mutational analyses (Melegari et al., 2005). A recent study found additional vital phosphorylated sites (T160, S168, and S176) also contributed to pgRNA encapsidation, minus- and plus-strand DNA synthesis, and rcDNA synthesis (Jung et al., 2014). Despite several host cell kinases such as protein kinase C (PKC) (Kann and Gerlich, 1994; Wittkop et al., 2010), a 46-kDa serine protein kinase (Kau and Ting, 1998), and glyceraldehyde-3-phosphate dehydrogenase protein kinase (GAPD-PK) (Duclos-Vallee et al., 1998) have been discovered to phosphorylate the core protein *in vitro* in the past, none of these papers mapped phosphorylation to specific sites in HBc. Until Daub et al demonstrated that SR protein-specific kinases 1 and 2 (SRPK1 and SRPK2), which are approximately 95 and 115 kDa, respectively, were identified as the important kinases phosphorylating the core protein *in vitro*, as those serine phosphorylation sites (S155, S162, and S170) are SPRRR motifs in the CTD (Daub et al., 2002). However, the role of the SRPK-regulated phosphorylation to HBV replication is still confused. The reason is that another group found a new HBV replication pathway mediated by SRPK1 and SRPK2, which is independent of core protein phosphorylation (Zheng et al., 2005). These results hint that SRPKs have other functions which may affect HBV replication. Cyclin-dependent kinase 2 (CDK2), another controversial kinase targeting the core protein, was reported by Ludgate et al. (2012) CDK2 can both indeed phosphorylate the HBc CTD *in vitro* and *in vivo* because S/T-P motifs, which are specific sites for proline-directed serine-threonine kinases, are present in HBc. Ironically, the inhibitors of CDK2 had no apparent effect on HBV replication (Ludgate et al., 2012). The complicated relationship between host kinases may be the main reason that the inhibitors become invalid. A current report showed that polo-like-kinase 1 (PLK1), a host kinase involved in cell cycle regulation, can effectively promote HBV replication via directly binding with HBc, leading to phosphorylation of the latter (Diab et al., 2017). The authors also mapped the accurate phosphorylated sites to S168, S176, and S178 in the core protein (Diab et al., 2017). Progressive experiments suggested that viral replication can be effectively blocked through inhibiting PLK1 expression in the liver humanized mouse model (Diab et al., 2017). Moreover, they observed that alanine substitutions at the 3 S/P sites induce the loss of PLK1 phosphorylation. This result points out that CDK2 may play a priming role in HBc phosphorylation which promotes the subsequent phosphorylation by PLK1 (Diab et al., 2017). Although the kinases that phosphorylate the core protein are still undetermined, the combination of CDK2 and PLK1 is currently deemed to be more promising based on the above results.

**FIGURE 1:**
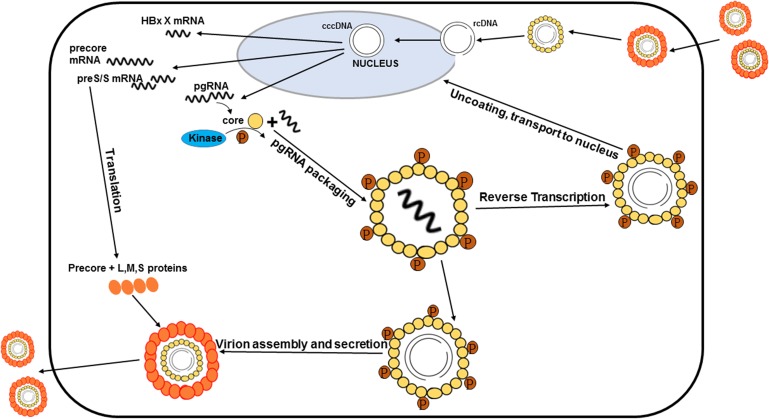
Processes of the HBV life cycle regulated by HBc phosphorylation. HBc phosphorylation can regulate reverse transcription, pgRNA packaging, subcellular localization, and virion secretion. Phosphorylation of the core protein is also essential for capsid maturation.

It is well-known that phosphorylation of the HBV core protein (HBc) is a crucial step during HBV lifecycle (Figure 1). HBc phosphorylation can regulate reverse transcription, pgRNA packaging, DNA synthesis, subcellular localization, and virion secretion (Lan et al., 1999; Gazina et al., 2000; Perlman et al., 2005; Basagoudanavar et al., 2007; Chua et al., 2010; Su et al., 2016). Phosphorylation of the core protein is also essential for capsid maturation and stability (Perlman et al., 2005; Basagoudanavar et al., 2007; Ludgate et al., 2016; Su et al., 2016). Moreover, HBc phosphorylation is implicated into regulating the chaperone activity of CTD (Chu et al., 2014).

In addition to viral protein phosphorylation, phosphorylation of the host factors can also influence the process of viral replication. For example, Hu et al. (2018) found that the inhibitory effect of SAMHD1 (a restriction factor of HBV) on HBV replication is abrogated when SAMHD1 is phosphorylated by CDK2.

HBc dephosphorylation is thought to be associated with nucleocapsid maturation (Perlman et al., 2005; Basagoudanavar et al., 2007). Perlman et al. (2005) suggested that the nucleocapsid shell is formed by the complete dephosphorylation of the core CTD. Further research by Basagoudanavar et al. (2007) demonstrated that subsequent core protein dephosphorylation at T239, S245, and S259 after minus-strand DNA synthesis can induce nucleocapsid maturation as well as facilitate the synthesis of the plus-strand DNA in duck HBV. A recent study clarified that core protein dephosphorylation appears in pgRNA assembly through a progressive immunoblotting assay (Zhao et al., 2018). A potential reason why core protein allosteric modulators (CpAMs), molecules that bind to the interfaces between the core protein dimer-dimer, can block pgRNA encapsidation is due to interference in core protein dephosphorylation during nucleocapsid assembly (Zhao et al., 2018). Further research demonstrated that core CTD dephosphorylation is not essential for subsequent nucleocapsid and envelope interactions that secrete virions via a mutagenesis assay (Ning et al., 2017). However, evidence that a direct role for triggering core protein dephosphorylation, nucleocapsid maturation and second-strand DNA synthesis is still lacking.

## The Role of Glycosylation in the Regulation of HBV Life Cycle

Glycosylation is another classical PTM type in controlling viral biological processes, specifically viral protein stability, virion secretion, and regulating host immune response (Spiro, 2002; Helenius and Aebi, 2004; Vigerust and Shepherd, 2007). Different glycosyltransferases and glycosidases are key factors for regulating protein glycosylation. Previous studies have demonstrated that *N*-glycosylation of HBV surface proteins, including large, middle, and small proteins, is crucial for virion secretion. In detail, the L and S proteins are necessary for virion formation, and increasing M protein expression levels is beneficial for virion secretion (Garcia T. et al., 2009; Ito et al., 2010). There is a single N-glycosylated site at N146 in the S domain that is essential for virion secretion. Mutations at either N146Q or N146S can block virion secretion, though subviral particle secretion is not affected (Ito et al., 2010). Mutations in N-glycosylated sites contribute to viral immune escape in light of harboring mutations within the HBsAg major hydrophilic region (Lambert and Prange, 2007; Liu et al., 2007; Ito et al., 2010). Progressively, researchers found out that mutations in the S protein provide a higher capacity to regulate virion secretion than those in the L/M protein (Ito et al., 2010; Bi and Tong, 2018). For example, the M133T mutation in the S protein can rescue the inhibition of virion secretion induced by the classic immune escape mutants G145R (El Chaar et al., 2010; Locarnini and Yuen, 2010) and R169H (Ito et al., 2010; Bi and Tong, 2018). To explain this phenomenon, authors found the additional higher molecular weights envelope proteins are associated with the presence of the M113T mutation by Western blot analysis (Ito et al., 2010). They subsequently proved that a new N-linked glycosylation site at N131 (^131^NST^133^) is created by the M133T mutation in the S protein. This novel site will produce additional forms of S (gp30), M (gp39), and L (gp45) proteins which are all essential for virion formation and secretion (Ito et al., 2010).

In addition, *O*-glycosylation of HBV surface proteins has been reported (Tolle et al., 1998; Werr and Prange, 1998; Schmitt et al., 1999; Tai et al., 2002). *O*-glycosylation of HBV surface proteins varies from different HBV genotypes. Researchers found that *O*-glycosylation appears in the M protein but not the L protein of HBV genotype D (Tolle et al., 1998; Schmitt et al., 1999). Potential *O*-glycosylation motifs are also found in HBV genotype B, C, E, and F except genotype A (Tai et al., 2002). Tai et al. (2002) reported that *O*-glycosylation in the preS2 region can partially cause size heterogeneity of wild type M protein. They suggested that enhanced *O*-glycosylation may be associated with hypermodification of the internally deleted M protein leading to the immune escaping (Tai et al., 2002).

Glycosylation also affects HBV entry into hepatoma cells. A recent study stated that core-fucosylation plays an important role in HBV infection through HBV-receptor-mediated endocytosis in a pseudovirus HBV model (Takamatsu et al., 2016). From their observations, sodium taurocholate cotransporting polypeptide (NTCP), a classical HBV receptor, is coprecipitated with other proteins and relied on core-fucosylation levels (Takamatsu et al., 2016). In contrast, an opinion from another recent study demonstrated that *N*-glycosylation is not needed for NTCP to mediate HBV infection in an *in vitro* experiment (Lee et al., 2018). The authors observed that HepG2 cells are glycosylated at two relative glycosylation sites, N5 and N11, when NTCP is introduced, and mutating either or both sites is unable to block cell surface NTCP availability and its subcellular localization, leading to the inability to inhibit HBV infection (Lee et al., 2018). Thus, further research is essential to explain these controversial results.

## The Role of Acetylation in the Regulation of HBV Life Cycle

**FIGURE 2:**
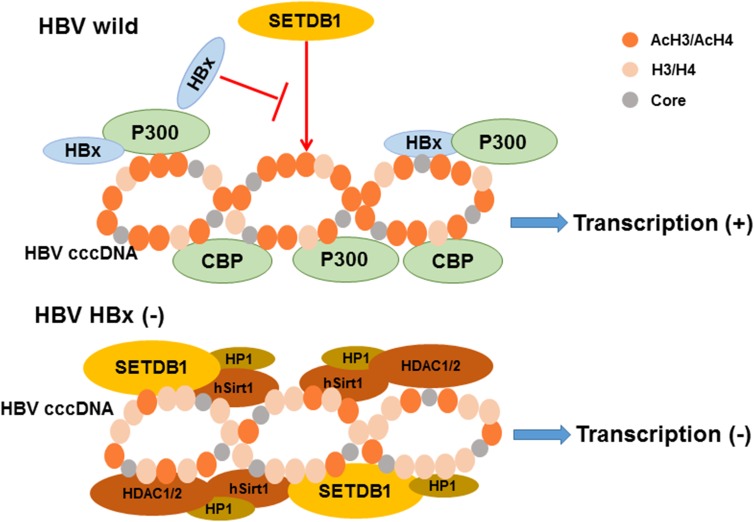
The model of relevant chromatin modifying enzymes involved in the regulation of HBV cccDNA transcription influenced by HBx. As indicated, the recruitment of chromatin modifying enzymes onto cccDNA is modulated by HBx expression. The activation of histone acetylation is caused by the interaction of HBx and acetyltransferase p300/CBP complex leading to the cccDNA transcription. In contrast, the recruitment of the histone deacetylases hSirt1 and HDAC1/2 are increased induce silencing of cccDNA transcription in the absence of HBx expression. Besides, hypoacetylation is also accompanied by the recruitment of heterochromatin protein 1 factor (HP1) and SET domain bifurcated 1 (SETDB1). Modified from Riviere et al. (2015).

Acetylation of lysine, which modifies protein primary properties, has a role in various biological processes including viral proliferation and chromatin remodeling (Choudhary et al., 2009; Deribe et al., 2010). It is inversely regulated by acetyltransferases and deacetylases. It has been reported that histone acetylation is implicated in the activation of replication and transcription due to its influences on histones and histone interactions (Strahl and Allis, 2000). The template for the transcription of HBV mRNA, cccDNA, is formed into minichromosomes by histone and non-histone proteins. Researchers found that HBV replication is regulated by the acetylation of cccDNA-bound H3 and H4 histones based on ChIP experiments in 2006 (Pollicino et al., 2006). The application of histone deacetylase inhibitors induces an increase in both HBV replication and cccDNA-bound acetylated H4 (Pollicino et al., 2006). They also revealed that low HBV replication in patients is correlated with the recruitment of p300/CBP and histone deacetylases 1 (HDAC1) to cccDNA (Pollicino et al., 2006). Additional research by another group demonstrated that the processing of relevant enzymes involved in viral minichromosome formation is modulated by HBx (Belloni et al., 2009). The proof that preventing cccDNA deacetylation is regulated by HBx was shown by an evident increase in the histone deacetylases hSirt1 and HDAC1 due to an HBx mutant (Belloni et al., 2009). Previous studies showed that HBx can directly interact with the acetyltransferase p300/CBP complex to promote transcription, causing the activation of the acetylated histone state in HBV minichromosomes (Cougot et al., 2007; Zheng et al., 2009), which indicates that the balance of histone acetylation modifications in HBV is influenced by HBx (Figure 2). A recent study reported that IL6 treatment to NTCP-HepG2 cells causes a decrease in both cccDNA-bound histone acetylation and 3.5-kb pgRNA, leading to replication repression (Palumbo et al., 2015). In detail, the levels of H3 histone-bound cccDNA is slightly increased after IL-6 treatment; however, cccDNA-bound histones are predominantly hypoacetylated (Palumbo et al., 2015). Another new regulator of histone acetylation in HBV replication was discovered by Nishitsuji’s group. They explored how TIP60 (a catalytic subunit in the NuA4 complex localized in the cccDNA chromatin complex) catalyzed the acetylation of histone H4 to recruit Brd4, inducing the inhibition of HBV replication (Nishitsuji et al., 2018).

## The Role of Methylation in the Regulation of HBV Life Cycle

Protein methylation, inversely regulated by methyltransferases and demethylases (similar to acetylation), contributes to regulating a series of cellular biological processes, including proliferation, transcription, RNA metabolism, and chromatin remodeling (Nicholson et al., 2009; Deribe et al., 2010). It is well-established that PRMT1, a typical member of the protein arginine methyltransferase (PRMT) family, participates in HBV transcription (Benhenda et al., 2013). Researchers found that PRMT1 can negatively regulate HBV transcription via its methyltransferase activity, which was mainly reflected by histone H4 methylation (Benhenda et al., 2013). However, the inhibitory activity is dependent on the expression of HBx due to the interaction between PRMT1 and HBx (Benhenda et al., 2013). PRMT5, another PRMT, was previously reported in the repression of HIV long terminal repeat transcription through the methylation of SPT5, which is also a crucial HBV transcription regulator (Kwak et al., 2003). A recent study demonstrated that PRMT5 promotes cccDNA-bound H4R3me2s activity to inhibit cccDNA transcription by relying on its methyltransferase activity, while inhibition of core particle DNA production is methyltransferase-independent by preventing interactions with pgRNA (Zhang et al., 2017). Moreover, the interaction between PRMT5 and HBc was also demonstrated by CoIP assay in Huh7 cells (Zhang et al., 2017). Several months later, a group from the Czech Republic showed that PRMT5 not only interacts with HBc, but that PRMT3 also weakly binds with HBc by GST pull-down assay from HEK293T cells (Lubyova et al., 2017). They also found that HBc is methylated on its arginine residues at the C-terminal, with the R150 and R156 methylated sites being detected by mass spectrometry (Lubyova et al., 2017). The potential effect of arginine methylation in HBc is referred to as a regulator for controlling HBc binding to cellular factors, which participate in HBc shuttling between the nucleus and cytoplasm (Lubyova et al., 2017). Some studies have suggested that similar to the influence of histone acetylation, HBV cccDNA transcription is also regulated by histone methylation (Riviere et al., 2015; Ren et al., 2018). Plenty of previous studies have shown that methylation of H3 on lysine 9 (H3K9me) is controlled by histone lysine methyltransferases (HKMT), such as SETDB1 and SETD1A (Wang et al., 2003; Bannister and Kouzarides, 2011; Binda, 2013). A study by a group of French scientists demonstrated that HBV transcriptional silencing is related to H3K9me3 by ChIP-qPCR. The depletion of SETDB1 among the candidate HKMT promotes an obvious decrease in H3K9me3 (Riviere et al., 2015). Moreover, this process is controlled by HBx (Riviere et al., 2015). A similar mechanism was also presented for SETD1A in HBV transcription regulation in a recent paper (Ren et al., 2018).

Despite the number of studies on HBV transcription and replication regulation by methyltransferases, the role of demethylase involvement in the HBV life cycle is still obscure.

## The Role of Ubiquitination, Sumoylation and Neddylation in the Regulation of HBV Life Cycle

Proteins are modified by covalent bonding with various short modulated proteins, such as ubiquitin, small ubiquitin-like modifier (SUMO) or other ubiquitin-like proteins, under certain conditions in biological processes, such as transcription, protein secretion, and so on (Hershko and Ciechanover, 1998). Ubiquitination is mainly regulated by E1, E2, and E3 enzymes or negatively controlled by deubiquitination enzymes (DUBs). Focus on ubiquitination involved in the viral life cycle has been increasing. An earlier research reported that γ2-adaptin, a ubiquitin-interacting adaptor, is essential for HBV viral particle release in light of the interaction with the HBV core protein lysine residue 96 in a ubiquitin-dependent manner (Rost et al., 2006), indicating that core protein ubiquitination may be crucial in HBV release. Subsequently, the authors found that Nedd4, an ubiquitin ligating enzyme with C-terminal E3 ubiquitin ligase domains, can partially interact with the core protein due to the PPAY-like motif binding site in the core protein and induce HBV production (Rost et al., 2006). Although two potential ubiquitin acceptor lysine residues (K7 and K96) in the core protein have been discovered, K96, not K7, can serve as the ubiquitin binding site that induces core particle release. The direct evidence that core ubiquitination occurred is still poor and the potential mechanism whereby γ2-adaptin binds Nedd4 to regulate viral particle release has not been fully elucidated (Rost et al., 2006). However, an article suggested that the production of the HBV core particle is not controlled by direct ubiquitin binding to either K7 or K96 (Garcia M.L. et al., 2009). Therefore, no direct evidence of core protein ubiquitination is provided by these studies. Np95/ICBP90-like RING finger protein (NIRF), an E3 ligase, is known as a crucial regulator in cell proliferation (Iwata et al., 2009). A Chinese group discovered that NIRF can directly interact with the HBV core protein *in vivo* and promote its ubiquitination and degradation through E3 ubiquitin ligase activity (Qian et al., 2012). Additionally, Mature core particle release is also influenced by core protein proteasome degradation regulated by NIRF (Qian et al., 2012).

It is well-established that HBx interacts with damage-specific DNA binding protein 1 (DDB1) to regulate viral replication (Sitterlin et al., 1997, 2000; Bontron et al., 2002). DDB1 is also known as a linker protein for the assembly of Cullin 4-ROC1 RING E3 ubiquitin ligase (CRL4) complexes (Angers et al., 2006; He et al., 2006). In a recent paper, researchers found that the host restriction factor SMC5/6 (a direct ubiquitination substrate of the HBx-CRL4 complex) can inhibit HBV replication. HBx can degrade SMC5/6, leading to enhanced HBV gene expression in a proteasome-dependent manner (Murphy et al., 2016). Therefore, this study provides evidence that HBx may promote HBV replication by degrading host restriction factors through the cullin ubiquitin E3 ligase, which requires interactions between DDB1 and HBx. A contrasting result from a South Korean group was that DDB1 itself can stimulate HBV transcription in an HBx-independent manner (Kim et al., 2016). However, the mechanism by which DDB1 promotes transcription with a cccDNA template has not been elucidated.

SUMOylation is a reversible PTM modulated by small ubiquitin-related modifier (SUMO) proteins, such as SUMO1 and SUMO2/3. SUMOylation is a crucial regulator of protein stability and subcellular localization and interactions (Zhao, 2018). Previous studies have shown that SUMOylation can regulate target protein stability by interfering with ubiquitination (Chen S. et al., 2013; Citro et al., 2013). In a recent study, SUMOylation was reported for its potential influence on HBV ability to evade host immune response (Sengupta et al., 2017). In detail, promyelocytic leukemia nuclear bodies (PML-NB) protein Speckled 110 kDa (Sp110) undergoes a deSUMOylation-driven release from PML-NBs to promote HBV proliferation and can also interact with HBx to maintain HBx stability and modulate several direct target genes of HBx transcription, ultimately inducing persistent viral infection (Sengupta et al., 2017).

NEDDylation is also a reversible PTM that is mainly modulated by the ubiquitin-like molecule neuronal precursor cell-expressed developmentally down regulated protein 8 (NEDD8) via a three-step enzymatic reaction catalyzed by NEDD8-activating enzyme E1 (NAE), NEDD8-conjugating enzyme E2 (UBC12/UBE2M) and NEDD8-E3 ligases (Zhou et al., 2018). Similar to SUMOylation, NEDDylation can also regulate diverse signaling pathways, such as apoptosis, autophagy, and inflammatory responses, by modulating protein stability and interactions (Zhou et al., 2018). A recent study discovered that HBx can be stabilized by the E3 ligase HDM2 in a NEDDylation-dependent manner at Lys91 and Lys95 (Liu et al., 2017). NEDDylated HBx can effectively prevent interactions with the E3 ligase Siah-1, leading to inhibited HBx degradation. Moreover, HBx NEDDylation can promote HBx chromatin localization and regulate the transcription of downstream genes, such as IL-8 and YAP, to promote cell proliferation and hepatocarcinogenesis (Liu et al., 2017).

## The Role of PTMs in Hepatocarcinogenesis Due to HBV

It is well-known that major HBV mechanisms related to hepatocarcinogenesis include viral integration and the regulation of trans-activating HBV proteins, such as HBx (Park et al., 2013). HBx can associate with the androgen receptor (AR) signaling pathway, a classical pathway that contributes to HCC, to promote hepatocarcinogenesis (Chiu et al., 2007). Subsequent research has demonstrated that the enhancement of AR transcriptional activity results from AR N-terminal transactivation domain (NTD) phosphorylation by c-Src kinase, which is indirectly activated by HBx (Yang et al., 2009). Moreover, the upstream element cell cycle-related kinase (CCRK) can also control HBx expression through the phosphorylation of glycogen synthase kinase 3β (GSK3β) (Yu et al., 2014).

Regulation by protein phosphorylation, ubiquitination and SUMOylation is also involved in another classical signaling pathway associated with HBx, the NF-κB pathway, in the pathogenesis of HBV-induced HCC. Akt can phosphorylate IKKα at Thr23 and promote its ubiquitination in response to HBx overexpression (Huang et al., 2012). A recent study reported that p65, a crucial NF-κB subunit, can be stabilized through SUMOlyation interactions with SUMO2/3 (Liu J. et al., 2015). In another current study, SUMOylated centrosomal P4.1-associated protein (CPAP) was reported to be implicated in IKK-mediated NF-κB activation, leading to enhanced HBx-induced NF-κB signaling in HBV-related HCC (Yang et al., 2013). HBx also promotes the up regulation of MSL2, which induces APOBEC3B degradation through ubiquitination to maintain HBV cccDNA stability, thus contributing to hepatocarcinogenesis (Gao et al., 2017). Additionally, Li et al. (2018) demonstrated that HBx can induce the phosphorylation of PDK1, in turn to activate the PDK1-WINK1 pathway in HBV-associated HCC tissues. The authors also discussed that activation of the PDK1-WINK1 pathway mediated by HBx may be implicated in the development of HBV-associated HCC (Li et al., 2018).

Although plenty of studies have demonstrated that HBx may indirectly influence protein PTMs leading to hepatocarcinogenesis, HBx can also interfere with protein PTMs through direct interactions. For example, HBx can directly interact with amplified in breast cancer 1 (AIB1), a crucial enhancer required for the activation of specific transcription factors, such as NF-kB and AP-1, preventing interactions between Fbw7α and AIB1 and inhibiting the Fbw7α-mediated ubiquitination and degradation of AIB1, leading to HCC cell invasiveness (Liu et al., 2012). Another example of an HBx direct interaction is that HBx can stabilize the Myc oncoprotein by preventing the interaction between Myc and the Skp/cullin/F-box (SCF) ubiquitin E3 ligase to block Myc oncoprotein ubiquitination and degradation, which ultimately contributes to viral oncogenesis (Lee et al., 2016).

## The Role of PTMs in Host Antiviral Resistance Due to HBV

The interferon system is involved in host antiviral responses. Type I interferons (IFNs) exhibit their antiviral functions according to the activation of the Janus kinase-signal transducer and activator of transcription (JAK-STAT) signaling pathway (Schneider et al., 2014). It is well-established that some host factors, induced after HBV infection, can regulate the activation of STAT1 via diverse PTMs (Christen et al., 2007; Chen J. et al., 2013, Chen K. et al., 2017; Bing et al., 2014). For instance, a previous study discovered that HBV induced the up regulation of serine/threonine phosphatase (PP2A), which can effectively block STAT1 activation due to inhibition of STAT1 arginine methylation by directly interacting with PRMT1 (Christen et al., 2007). Viral proteins also have been testified to inhibit STAT1 activation. A group found that HBV polymerase can repress the activation of STAT1 by blocking the association between STAT1 and protein kinase C-δ (PKC-δ), which can phosphorylate STAT1 Ser727, leading to its activation (Chen J. et al., 2013).

Additionally, modulation of the type I IFN receptor (IFNAR1) promotes its binding with IFN-α to activate the JAK-STAT pathway; this interaction also plays an important role in the interferon-mediated antiviral pathway. Researchers have recently determined that matrix metalloproteinase 9 (MMP-9), which originates from peripheral blood mononuclear cells (PBMCs) and macrophages, can be up regulated by HBV stimulation and interacts with the IFNAR1 extracellular domain to disrupt binding with IFN-α, subsequently facilitating the phosphorylation and ubiquitination of IFNAR1 in a p38 and Ser532 phosphorylation-independent manner (Chen J. et al., 2017).

The HBV-mediated disruption in IFN-β production is also related to PTMs on stimulator of interferon genes (STING), which has been reported to be crucial for the regulation of the immune response to cytosolic DNA (Liu Y. et al., 2015). Previous studies showed that STING ubiquitination is necessary for the induction of IFN-β (Tsuchida et al., 2010; Zhang et al., 2012). The results from Yuan’s group showed that HBV polymerase can physically associate with STING to inhibit STING K63-linked polyubiquitination, while the interactions with the E3 ubiquitin ligases TRIM32, TRIM56, and STING are not influenced (Liu Y. et al., 2015). Additionally, some crucial host factors that are induced by HBV can affect the IFN-β production via different PTMs. NF-κB essential modulator (NEMO) is involved in the process of IFN-β production through phosphorylating IFN regulatory factor 3 (IRF3) in a polyubiquitin-dependent manner (Clark et al., 2013). HBV-induced rubicon can directly bind to NEMO leading to inhibition of NEMO ubiquitination and further influencing the IFN-β production (Wan et al., 2017). Another research reported that parkin, a host factor induced by HBV infection, can modulate the linear ubiquitin chain on the MAVS (a mitochondrial membrane protein) signalosome, which abrogates IFN-β synthesis (Khan et al., 2016).

## Conclusion and Perspectives

In the past few decades, a growing number of studies have focused on the diverse functions and complicated mechanisms between PTMs and viral proteins, such as HBc (Table 1); or crucial host factors in controlling viral biological processes. However, the reasons behind specific phenomena and controversial experimental results are still obscure, demonstrating the need for further investigation. Even though phosphorylation, acetylation, methylation, and ubiquitination are increasingly shown to play important roles in HBV replication and transcription, other PTMs including succinylation, hydroxylation, nitration, and carbonylation also potentially participate in this regulation, showing that our understanding of these regulatory processes is still unclear. Aside from the classical function of some PTMs, such as glycosylation, other roles for PTMs in regulating HBV life cycle should be further explored. It has been mentioned that advanced technologies, including new types of mass spectrometry and elaborate analyses that consist of big data analysis, are required to discover novel proteins changes. Additionally, interactomics and fluxomics, new powerful omics studies focused on protein interactions research, should be applied to observe dynamic changes for crucial proteins in the HBV life cycle. In conclusion, further studies need to be performed to address unresolved or controversial problems associated with PTMs regulating mechanisms and potentially unknown PTMs. Identifying new biological functions will not only enrich the theoretical knowledge on PTMs regulating HBV biological processes but also contribute to the identification of effective diagnostic markers and therapeutic targets for diseases caused by HBV infection.

**Table 1:** Viral proteins related post-translational modification (PTM).

Viral proteins	PTM	Target proteins	Enzymes/ regulators (physical interactors)	Modification position in viral proteins	Contribution to HBV life cycle/pathogenesis of HBV related liver diseases	Reference
HBc	Phosphorylation	Yes	PKC	Not mentioned	HBc phosphorylation by PKC is essential for HBV envelopment. Genome maturation is not affected by PKC inhibition. PKC inhibition induces the failure of virion release.	Kann and Gerlich, 1994; Wittkop et al., 2010
HBc	Phosphorylation	Yes	A 46 kDa kinase	Not mentioned	HBc phosphorylation is crucial for pregenome encapsidation. The detailed function of this kinase is unknown.	Kau and Ting, 1998
HBc	Phosphorylation	Yes	GAPD-PK	Not mentioned	GAPD-PK can phosphorylate the core subunits. The detailed function of this kinase is unknown.	Duclos-Vallee et al., 1998
HBc	Phosphorylation	Yes	SRPK1/2	HBc S155, S162, S170	HBc phosphorylation by SRPK1/2 is essential for encapsidation step and required for nuclear import of the viral genome.	Daub et al., 2002
HBc	Phosphorylation	Yes	CDK2	HBc S155, S162, S170	HBc phosphorylation by CDK2 induces HBV capsids assembled. However, CDK2 inhibitors have no apparent effect on HBV replication.	Ludgate et al., 2012
HBc	Phosphorylation	Yes	PLK1	HBc S168, S176, S178	HBc directly interacts with PLK1. HBc phosphorylation is associated with HBV replication increasing. PLK1 inhibition induces suppression of HBV DNA accumulation. CDK2 may play a priming role in HBc phosphorylation which promotes the subsequent phosphorylation by PLK1.	Diab et al., 2017
HBc	Dephosphorylation	Yes	Unknown	Potential HBc T239, S245 and S259 (duck HBV)	HBc dephosphorylation is associated with nucleocapsid maturation, nucleocapsid shell is formed by the complete dephosphorylation of the HBc CTD and dephosphorylation is also crucial for pgRNA assembly.	Perlman et al., 2005; Basagoudanavar et al., 2007; Ning et al., 2017; Zhao et al., 2018
HBs	N-Glycosylation	Yes	N-glycosidase	N146 in the common S domain (wild type)	*N*-glycosylation of HBV surface proteins is crucial for virion secretion. In detail, the L and S proteins are necessary for virion formation, and M protein is essential for virion secretion. Mutation in N-glycosylated site may benefit for HBV immune escaping.	El Chaar et al., 2010; Ito et al., 2010
HBs	O-Glycosylation	Yes	O-glycosidase	C-terminal pre-S2 region of M protein, potential Thr-37 (but not in HBV genotype A)	*O*-glycosylation of HBV surface proteins varies from different HBV genotypes. *O*-glycosylation in the preS2 region can partially cause size heterogeneity of wild type M protein.	Tolle et al., 1998; Werr and Prange, 1998; Schmitt et al., 1999; Tai et al., 2002
HBx	Acetylation	No	p300/CBP complex	Not target protein	HBx directly interacts with the acetyltransferase p300/CBP complex to promote HBV transcription, leading to the activation of the acetylated histone state in HBV minichromosomes.	Cougot et al., 2007; Zheng et al., 2009
HBx	Methylation	No	PRMT1	Not target protein	HBx directly interacts with PRMT1, inhibits PRMT1 methyltransferase activity to enhancing HBV transcription.	Benhenda et al., 2013
HBc	Methylation	Yes	PRMT3, PRMT5	Potential HBc R150 and R156	PRMT3 weakly binds to HBc. PRMT5 directly interacts with HBc. HBc methylation is referred to as a regulator for controlling HBc binding to cellular factors, which participate in HBc shuttling between the nucleus and cytoplasm.	Lubyova et al., 2017; Zhang et al., 2017
HBc	Ubiquitination	Yes	Nedd4	Potential HBc K96 (unproved)	Nedd4 partially interacts with HBc, promotes HBV maturation and release.	Rost et al., 2006
HBc	Ubiquitination	Yes	NIRF	Not mentioned	NIRF directly interacts with HBc, promotes HBc degradation, leading to HBV replication inhibition.	Qian et al., 2012
HBx	Ubiquitination	No	CRL4 E3 ligase	Not target protein	HBx directly interacts with CRL4 E3 ligase, promotes the ubiquitination and degradation of SMC5/6 to enhancing HBV replication.	Murphy et al., 2016
HBx	SUMOylation	No	Sp110	Not target protein	HBx directly interacts with Sp110, drives it out of the PML-NB to inhibiting SUMOylation of Sp110, and induces viral persistence.	Sengupta et al., 2017
HBx	NEDDylation	Yes	E3 ligase HDM2	HBx Lys91 and Lys95	HDM2 promotes HBx stabilization by NEDDylation. NEDDylation of HBx facilitates its chromatin localization, transcriptional regulation activity and its function in tumor growth.	Liu et al., 2017
HBx	Ubiquitination	No	AIB1	Not target protein	HBx directly interacts with AIB1, inhibits Fbw7α-mediated ubiquitination of AIB1, and cooperates with AIB1 to promote HCC via enhancing MMP-9 expression.	Liu et al., 2012
HBx	Ubiquitination	No	Myc	Not target protein	HBx directly binds to Myc, inhibits Skp2-mediated Myc ubiquitination, and contributes to hepatocarcinogenesis through Myc stabilization.	Lee et al., 2016
HBV polymerase	Phosphorylation	No	PKC-δ	Not target protein	The terminal protein (TP) and RNase H (RH) domain of HBV polymerase directly binds to PKC-δ, inhibits STAT1 Ser727 phosphorylation, inducing the inhibition of IFN-α signaling activation.	Chen K. et al., 2017
HBV polymerase	Ubiquitination	No	STING	Not target protein	The reverse transcriptase (RT) and RNase H (RH) domain of HBV polymerase directly binds to STING, inhibits K63-linker polyubiquitination of STING, leading to the inhibition of IFN-β production.	Liu Y. et al., 2015

## Author Contributions

FY wrote the whole manuscript and prepared the table.

## Conflict of Interest Statement

The author declares that the research was conducted in the absence of any commercial or financial relationships that could be construed as a potential conflict of interest.
